# Autonomic dysfunction in post-COVID patients with and witfhout neurological symptoms: a prospective multidomain observational study

**DOI:** 10.1007/s00415-021-10735-y

**Published:** 2021-08-12

**Authors:** Alex Buoite Stella, Giovanni Furlanis, Nicolò Arjuna Frezza, Romina Valentinotti, Milos Ajcevic, Paolo Manganotti

**Affiliations:** 1 Clinical Unit of Neurology, Department of Medicine, Surgery and Health Sciences, Trieste University Hospital-ASUGI, University of Trieste, Strada di Fiume, 447, 34149 Trieste, Italy; 2 School of Medicine and Surgery, Department of Medicine, Surgery and Health Sciences, Trieste University Hospital-ASUGI, University of Trieste, Strada di Fiume, 447, 34149 Trieste, Italy; 3 Infectious Diseases, Trieste University Hospital-ASUGI, University of Trieste, Strada di Fiume, 447, 34149 Trieste, Italy; 4 Department of Engineering and Architecture, University of Trieste, Via Alfonso Valerio 10, Trieste, Italy

**Keywords:** COVID-19, Long-COVID, Autonomic dysfunction, COMPASS-31, Orthostatic intolerance

## Abstract

The autonomic nervous system (ANS) can be affected by COVID-19, and dysautonomia may be a possible complication in post-COVID individuals. Orthostatic hypotension (OH) and postural tachycardia syndrome (POTS) have been suggested to be common after SARS-CoV-2 infection, but other components of ANS function may be also impaired. The Composite Autonomic Symptom Scale 31 (COMPASS-31) questionnaire is a simple and validated tool to assess dysautonomic symptoms. The aim of the present study was to administer the COMPASS-31 questionnaire to a sample of post-COVID patients with and without neurological complaints. Participants were recruited among the post-COVID ambulatory services for follow-up evaluation between 4 weeks and 9 months from COVID-19 symptoms onset. Participants were asked to complete the COMPASS-31 questionnaire referring to the period after COVID-19 disease. Heart rate and blood pressure were manually taken during an active stand test for OH and POTS diagnosis. One-hundred and eighty participants were included in the analysis (70.6% females, 51 ± 13 years), and OH was found in 13.8% of the subjects. Median COMPASS-31 score was 17.6 (6.9–31.4), with the most affected domains being orthostatic intolerance, sudomotor, gastrointestinal and pupillomotor dysfunction. A higher COMPASS-31 score was found in those with neurological symptoms (*p *< 0.01), due to more severe orthostatic intolerance symptoms (*p *< 0.01), although gastrointestinal (*p *< 0.01), urinary (*p *< 0.01), and pupillomotor (*p *< 0.01) domains were more represented in the non-neurological symptoms group. This study confirms the importance of monitoring ANS symptoms as a possible complication of COVID-19 disease that may persist in the post-acute period.

## Introduction

Novel coronavirus disease (COVID-19) has been suggested to not only affect health during the acute phase of the SARS-CoV-2 infection, but some manifestations have been consistently reported in several studies also after the recovery. Such phenomenon has been named “long-COVID” or “post-acute COVID-19” depending on the time course definition of the persisting or novel symptoms [1], the latter being defined as a syndrome characterized by persistent symptoms and/or delayed or long-term complications beyond 4 weeks from the onset of COVID-19 [2]. These complications include myocarditis, pulmonary fibrosis, encephalitis, thromboembolic events, psychiatric illness, and persisting symptoms such as dyspnea, cough, and fatigue [3, 4]. Among these clinical manifestations, neurological symptoms may be also common, as cognitive deficits have been reported in 36% of patients two to four months after COVID-19 [5, 6], and the reported fatigue and dysexecutive syndrome may depend on cortical reorganization [7]. Despite few data are present and further investigations are recommended, age, higher BMI and female sex have been proposed as risk factors for post-COVID [8]. Some recent papers have pointed a rationale for autonomic dysfunction following COVID-19, suggesting a role of the virus infection and/or of the related immune response on the autonomic nervous system (ANS), often resulting in orthostatic intolerance (OI), including orthostatic hypotension (OH) and postural tachycardia syndrome (POTS) [9, 10]. OH and POTS can be common in the elderly, particularly in geriatrics outpatients [11], as well as in individuals with other dysautonomic features like in neurological diseases (e.g., Parkinson or dementia) [12] or diabetes [13], the latter due to the possible presence of neuropathies affecting baroceptors and increased arterial stiffness [13, 14]. Such symptoms are often disabling and affect people’s quality of life; additionally, they can increase the risk of further events and falls. A recent case series reported typical features of orthostatic intolerance, fatigue, and activity intolerance in post-COVID individuals, and autonomic testing found heterogeneous responses, including OH and POTS [15]. As of this time, reports of COVID-19-related dysautonomia have been limited, but some pathophysiological mechanisms have been suggested, as para- or post-infectious immune-mediated process [15], supported by the presence of dysautonomia in Guillain-Barré syndrome and acute autoimmune autonomic neuropathy [16, 17]. For these reasons, patients with persisting or novel symptoms after COVID-19 should be assessed for autonomic disorders, including an active stand test measuring blood pressure and heart rate [8, 9] following standard procedures [18]. Due to the hypothesized influence of SARS-CoV-2 infection on the autonomic nervous system, dysautonomia manifestations other than orthostatic syndrome might be supposed. The Composite Autonomic Symptom Scale 31 (COMPASS-31) questionnaire is a widely validated tool to assess symptoms of ANS dysfunction, and it evaluates six domains related to ANS function: OI, vasomotor, secretomotor, gastrointestinal, urinary, and pupillomotor [19].

The aim of this study was to assess the prevalence of ANS dysfunction, evaluated with the COMPASS-31 questionnaire and an active stand test, in a real-life setting in consecutive patients referred to the post-COVID ambulatory service, and to compare the patients who presented neurological symptoms with those without neurological manifestations.

## Materials and methods

This prospective observational study included patients who referred to the post-COVID ambulatory service of the University Hospital and Health Services of Trieste (ASUGI) between the 15.02 and 15.05 of 2021. All the procedures were performed according to the Declaration of Helsinki and the local institutional review board and ethics committee (CEUR-FVG) approved the study. To be included in the study, participants had to present persistent symptoms and/or delayed or long-term complications between 4 weeks and 9 months from the onset of COVID-19 confirmed by a nasopharyngeal swab. Participants were excluded if before COVID-19 they suffered from ANS dysfunctions or cognitive impairment, or if they were prescribed medications that might alter ANS as: antidepressants, beta-blockers, ACE inhibitors, calcium channel blockers, alpha-1 blockers, antihistamines, cholinesterase inhibitors, and central antihypertensives. Those who reported a cognitive deficit as a post-COVID symptom received the Montreal Cognitive Assessment (MoCA) and neuropsychological evaluation, and if the corrected score was lower than 15 or the neuropsychological evaluation suggested a remarkable cognitive deficit, they were excluded from the analysis. Participants were grouped between those presenting post-COVID neurological symptoms based on the medical check performed by an infectious disease specialist working in the post-COVID ambulatory service and then confirmed by the neurologist according to the clinical and instrumental (where possible, e.g., nerve conduction study) evaluation.

### ANS dysfunction evaluation

During the medical check, all the participants who met the inclusion and exclusion criteria were invited to complete the validated Italian version of the COMPASS-31 questionnaire [20]. To better assess post-COVID autonomic dysfunction, all questions were adapted to refer to the period after COVID-19, and improvements/worsening were evaluated comparing the time of the visit with the time when the dysautonomic symptoms appeared. A pilot testing was conducted on 10 post-COVID patients (not included in this study) on two separate occasions 14 days apart, according to previous surveys development [21–23] to investigate the reliability of the adapted version of the questionnaire employing a test–retest correlation that confirmed the appropriate reliability (Pearson’s *r* > 0.900, *p *< 0.01). The total score of COMPASS-31 and domains score were computed according to the original instructions [19]. COMPASS-31 score was presented as a continuous variable (0–100) and a cutoff of 13.25 was used to suggest ANS dysfunction, as previously defined for diagnosis of multiple system atrophy with predominant parkinsonism [24]. Domains scores were presented as continuous variables; the maximum weighted scores for each subdomain are as follows: 40 for orthostatic intolerance, 5 for vasomotor dysfunction, 15 for secretomotor dysfunction, 25 for gastrointestinal (GI) dysfunction, 10 for urinary dysfunction, and 5 for pupillomotor dysfunction [19].

Although not present in the original COMPASS-31 questions, additional items were included for a better understanding of other possible features of ANS dysfunction following COVID-19. These questions included the subjective evaluation of (i) thermal/environmental comfort (patients were asked if after COVID-19 they felt more uncomfortable while staying in hot, cold, humid, or windy environments), (ii) heat and cold sensation (if they were more/less sensible to contact heat and cold), and (iii) sexual dysfunction (e.g., impotence and reduced lubrication). These items were scored with a yes/no answer.

The active stand test was performed at the end of the visit, following guidelines [18]. Measures were manually taken with a sphygmomanometer [25] by a trained neurologist in OI evaluation, asking the patient to rest in the supine position for 5 min before the first measure, and then 3 min after standing. OH was defined as a fall of > 20 mmHg systolic and > 10 mmHg diastolic after standing for 3 min [18], and corrected in case of supine hypertension [26], while POTS is characterized by orthostatic symptoms (in the absence of orthostatic hypotension) with an increase in heart rate of 30 beats per minute or more when standing for more than 30 s [18, 27].

### Statistical analysis

All analyses were conducted using IBM SPSS Statistics (v.22.0, Chicago, IL, USA). Kolmogorov–Smirnov test of normality was performed. Categorical variables are presented as counts and percentages (%), while normally distributed continuous variables are presented as means and standard deviations (SDs) and non-normally distributed continuous variables are presented as medians (25th–75th percentile). To determine whether the associations between disease features and severity and the total COMPASS-31 score resulted from global autonomic symptoms across multiple subdomains, we grouped patients by the number of positive subdomains (score > 0 in any category) of the COMPASS-31: (1) the “global autonomic symptoms” group included all patients with five or more positive subdomains, and (2) the “limited autonomic symptoms” group included patients with fewer than five positive subdomains [28]. Post-COVID patients who presented neurological symptoms were compared to post-COVID patients without neurological symptoms, and further subgroup analysis compared the neurological patients according to their prevalent symptom. Between-groups comparisons were performed with the Mann–Whitney *U* test and the Fisher’s exact test. A univariate analysis was performed to investigate the association of different factors, including age, sex, and clinical characteristics, with the COMPASS-31 score. Factors that were associated with this score with *p *< 0.10 were included in the stepwise multivariable model and were confirmed as significant if *p *< 0.05, as previously described [29–31]. A binary logistic regression was performed between the COMPASS-31 total score and the OI domain, with the presence of OH and POTS as evaluated during the stand test. A value of *p *< 0.05 was considered significant.

## Results

### Demographics and clinical characteristics of the sample

One-hundred and ninety-two patients were first included in data collection; after excluding those who had a remarkable cognitive impairment, 180 participants were included in the final analysis (70.6% females, 51 ± 13 years). Among these, 97 were characterized by neurological manifestations (myalgia/asthenia 22.7%, headache 13.4%, hyposmia/hypogeusia 37.1%, dizziness 7.2%, sleep disturbances 10.3%, “brain fog”/cognitive deficit 42.3%), while 83 did not presented neurological symptoms but reported other post-COVID complications (61.3% exertional dyspnea, 29.1% arthralgia, 9.6% other) (Table 1). The median time from COVID-19 onset to the visit was 59 (31–175 days).

**Table 1:** COMPASS-31 score, domains, and additional items in post-COVID patients with and without neurological symptoms

	Neuro *n *= 97	Others *n *= 83	Significance
Age (y)	53 ± 14	49 ± 11	0.073
Females [*n *(%)]	67 (69.1)	60 (72.3)	0.743
COMPASS-31			
Score [0–100]	20.7 (5.4–32.2)	14.2 (8.2–30.3)	**0.002**
> 13.25 [*n *(%)]	55 (56.7)	45 (55.4)	0.654
Orthostatic intolerance			
Score [0–40]	12.0 (0.0–20.0)	0.0 (0.0–20.0)	** < 0.001**
> 0 [*n *(%)]	53 (54.6)	39 (47.0)	0.369
Vasomotor			
Score [0–5]	0.0 (0.0–0.0)	0.0 (0.0–0.0)	0.226
> 0 [*n *(%)]	22 (22.7)	20 (24.1)	0.860
Secretomotor			
Score [0–15]	2.0 (0.0–6.0)	2.1 (0.0–6.4)	0.227
> 0 [*n *(%)]	53 (54.6)	45 (54.2)	1.000
Gastrointestinal			
Score [0–24]	3.0 (0.0–6.1)	4.5 (2.7–7.1)	** < 0.001**
> 0 [*n *(%)]	66 (68.0)	77 (92.8)	** < 0.001**
Urinary			
Score [0–10]	0.0 (0.0–1.0)	0.0 (0.0–1.1)	** < 0.001**
> 0 [*n *(%)]	26 (26.8)	38 (45.8)	**0.012**
Pupillomotor			
Score [0–6]	1.0 (0.0–2.0)	1.3 (0.3–2.3)	** < 0.001**
> 0 [*n *(%)]	59 (60.8)	64 (77.1)	**0.024**
Global autonomic symptoms [*n *(%)]	23 (23.7)	24 (28.9)	0.496
Environmental intolerance [*n *(%)]			
Heat	17 (17.5)	23 (27.7)	0.109
Cold	33 (34.0)	34 (41.0)	0.356
Humid	8 (8.2)	20 (24.1)	**0.004**
Wind	4 (4.1)	9 (10.8)	0.093
Subj. thermal sensation [*n *(%)]			
Heat	7 (7.2)	12 (14.5)	0.145
Cold	5 (5.2)	16 (19.3)	**0.005**
Sexual impairment [*n *(%)]	13 (13.4)	13 (15.7)	0.677

Means ± standard deviations, medians (25th–75th percentile) and proportions are reported

### ANS dysfunction

**Fig. 1:**
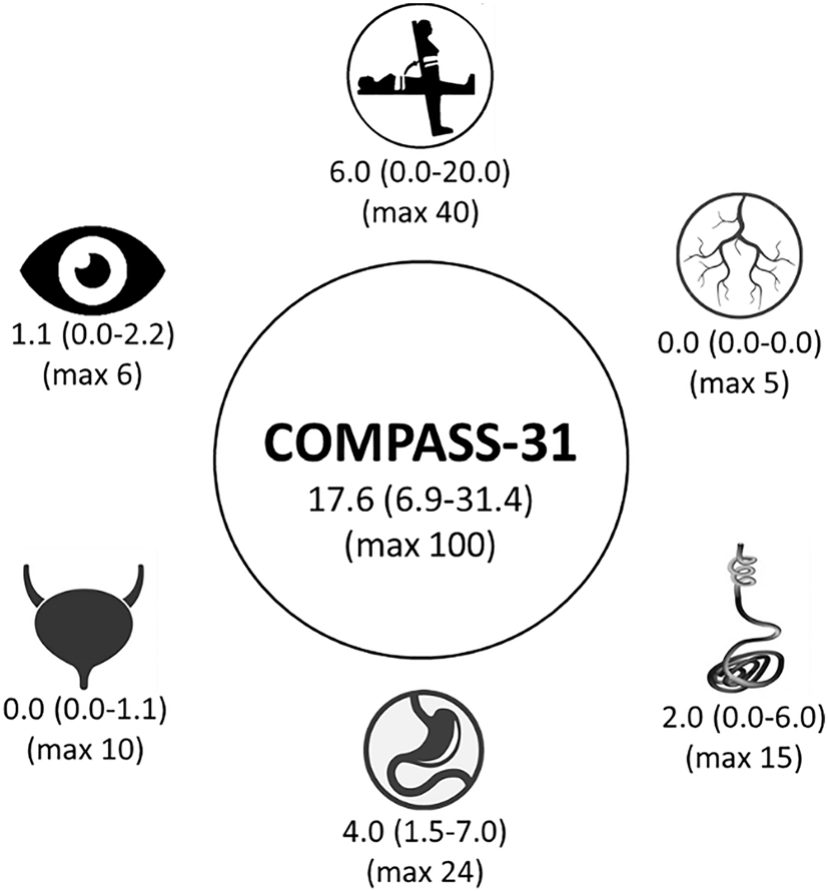
Graphical representation of median (25th–75th percentile) COMPASS-31 total score and subdomains scores, with maximal score for each domain, in post-COVID individuals (*n *= 180). From top, clockwise: orthostatic intolerance, vasomotor, sudomotor, gastrointestinal, urinary, pupillomotor domains

The median COMPASS-31 total score was 17.6 (6.9–31.4) and was higher than 13.25 in 61.1% of the sample (Fig. 1). The OI domain score was 6.0 (0.0–20.0) and was higher than 0 in 51.1% of the sample. The vasomotor domain score was 0.0 (0.0–0.0) and was higher than 0 in 23.3% of the sample. The secretomotor domain score was 2.0 (0.0–6.0) and was higher than 0 in 54.4% of the sample. The GI domain score was 4.0 (1.5–7.0) and was higher than 0 in 79.4% of the sample. The urinary domain score was 0.0 (0.0–1.1) and was higher than 0 in 35.6% of the sample. The pupillomotor domain score was 1.1 (0.0–2.2) and was higher than 0 in 68.3% of the sample. “Global autonomic symptoms” were found in 26.1% of the sample. Reduced tolerance to environmental conditions was found for heat (22.2%), cold (37.2%), humidity (15.6%) and wind (7.2%). Subjectively altered contact thermal sensation was found for heat (10.6%) and cold (11.7%). Sexual impairment was reported by 14.4% of the sample. Active stand test suggested the prevalence of OH in 13.8% of the sample, while POTS was found in none of the subjects. In particular, those with OH were mainly characterized by decreased diastolic blood pressure after 3 min standing (− 15 ± 3 mmHg).

**Fig. 2:**
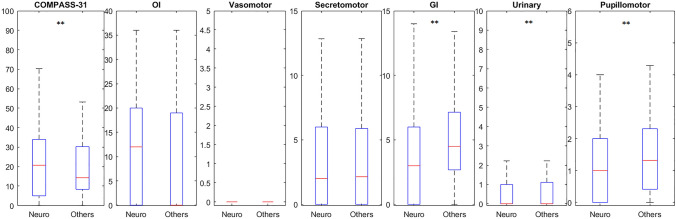
Boxplots showing the comparison between patients with neurological symptoms (neuro) and patients without neurological symptoms (others) at the time of the visit. COMPASS-31 score and subdomains with corresponding maximal score: orthostatic intolerance (OI), vasomotor, secretomotor, gastrointestinal (GI), urinary, and pupillomotor. **p < 0.01

Subgroups analysis between post-COVID patients with neurological symptoms and post-COVID patients without neurological symptoms (Table 1) suggested that, despite no significant differences in demographics or prevalence of ANS dysfunction (COMPASS-31 > 13.25), the post-COVID patients with neurological symptoms reported a higher overall COMPASS-31 score (*p *< 0.01) and OI score (*p *< 0.01), and less GI (*p *< 0.01), urinary (*p *< 0.05) and pupillomotor scores (*p *< 0.05) (Fig. 2). Similarly, intolerance to humidity (*p *< 0.01) and subjective cold sensation (*p *< 0.01) were less prevalent in the neurological symptoms group.

### Factors associated with ANS dysfunction

A trend for a negative association between age and COMPASS-31 score > 13.25 was found at the multivariate analysis (*p *< 0.072), suggesting younger subjects might present more dysautonomic symptoms, while sex was found significantly associated with COMPASS-31, suggesting females have a higher probability to develop ANS dysfunction (OR 2.771, 95% CI 1.091–7.035; *p *= 0.032). Binary logistic regression failed to show any association between the OI domain and OH assessed at the active stand test (*p *= 0.262). At the univariate analysis, both subjective altered heat sensation (OR 3.936, 95% CI 1.087–14.253; *p *= 0.39) and cold sensation (OR 4.609, 95% CI 1.289–16.477; *p *= 0.019) were significantly associated with COMPASS-31 score > 13.25. However, only cold sensation was significantly associated with the presence of positive responses in the OI domain (OR 4.007, 95% CI 1.271–12.631; *p *= 0.018). In the subgroup analysis among the patients with neurological symptoms at the visit, the regression analysis failed to show any specific association between the reported symptom(s) and ANS dysfunction or OH, and non-significant difference was found in the OI subdomain score between those with and without OH at the active stand test.

## Discussion

This study confirms and highlights the importance of assessing and monitoring ANS symptoms as possible long-term complications of SARS-CoV-2 infection. The main finding is the remarkable prevalence of ANS dysfunction in most post-COVID individuals, as shown by COMPASS-31 median and > 13.25 score, independent of the presence or absence of neurological symptoms. The most affected domains were orthostatic, secretomotor, urinary, and pupillomotor impairment, and these findings are in line with preliminary reports using COMPASS-31 in post-COVID patients [32], with a prevalence of a “global dysautonomia” in about 1/4 of the sample. Compared with previously published healthy controls from the literature [28, 33, 34], present data suggest a higher COMPASS-31 score in the post-COVID patients and ANS involvement. The absence of a significant association between age and COMPASS-31 score may depend on the specific questions asking for new dysautonomic symptoms after COVID-19, which might suggest an age-independent ANS response to the disease. Females were more commonly characterized by a higher COMPASS-31 score, in line with previous findings suggesting a higher prevalence of OI [35, 36] and symptomatic dysautonomia [37] in women. Comparison between patients with and without neurological complaining at the time of the visit suggested only minor differences, with a greater overall COMPASS-31 score in the neurological patients characterized by a higher OI domain score (despite no differences in the proportion of participants reporting at least one OI symptom) partially compensated by less GI, urinary and pupillomotor symptoms. Due to the absence of objective measures of autonomic function in the different domains, it is possible only to speculate that those with neurological symptoms post-COVID may suffer from more severe orthostatic impairment (despite some degree of lightheadedness may be present in all post-COVID participants), whereas those without neurological complications may be principally characterized by digestive and urinary alterations. The association between orthostatic impairment and neurological symptoms might be hypothesized due to the high prevalence of self-reported cognitive impairment that might be associated to altered postural vascular responses [38–40]. A common characteristic of the participants was the presence of fatigue, which can be a common post-COVID complication; however, no pathological differences between fatigued and non-fatigued patients on autonomic testing were previously found [41].

Despite few data have been published yet on dysautonomic symptoms and post-COVID, the rationale behind an ANS involvement during and after SARS-CoV-2 infection supports this study’s observations [9, 10, 42, 43]. Some of the mechanisms that have been hypothesized to influence ANS in COVID-19 include the sympathetic activation inducing pro-inflammatory cytokines that are at the base of the cytokine response [44]. As such, overactivation of the sympathetic nervous system may underlie the development of some responses that have been suggested to influence COVID-19 symptoms and recovery. In addition, the immune response may influence ANS function [45], as previously reported for OH and POTS being associated with antibodies (e.g., α-/β-adrenoceptors and muscarinic receptors), previous infections and autoimmune disorders [46–48]. Despite none of the patients was diagnosed with POTS, and OH was present in about one individual on 10, about one-half of the sample reported at least one symptom, although mild, of OI. This discrepancy, and the absence of a significant association between the OI component of COMPASS-31 and the active stand test, are not surprising: indeed, blood pressure and heart rate measurements were manually taken at a conventional time of 3 min, and this is known to potentially affect the assessment validity due to the measurement method and the different time courses of the responses [49]. Nevertheless, many questions still need to be clarified to understand the mechanisms underlying COVID-19 and vasovagal dysfunction, including the influence of SARS-CoV-2 on cerebral blood flow, possible hypoxia secondary to residual lung damage, prolonged bed rest, and the development of neuropathies [10, 50–55]. The present study included specific questions about subjective alterations in contact heat and cold sensations, which were reported to be altered in around 10% of the sample and were associated with higher COMPASS-31 total and OI domain scores. As such, these results support the hypothesis of a possible sensory or autonomic neuropathy involvement, although further studies including peripheral neurophysiology investigations are needed.

SARS-CoV-2 has been suggested to affect microcirculation, resulting in endothelial cell swelling and damage (endotheliitis), microscopic blood clots (microthrombosis), capillary congestion, and damage to pericytes, tissue repair angiogenesis, and scar formation [56]. In addition, a correlation is present between endothelial function and ANS [57], and vasomotor activity is a component of dysautonomia. However, symptoms of the vasomotor domain were the less commonly reported alteration in this sample (about 1/4), suggesting a minor impact of COVID-19 on vascular responses, or minor attention towards the related clinical manifestations (e.g., changes in hands and feet color).

About half of post-COVID individuals have reported secretomotor and sweating abnormalities. Together with the impaired environmental tolerance and thermal sensation, these observations suggest possible thermoregulatory alterations. However, since these findings were not supported by previously published objective measures (e.g., quantitative sensory testing—QST, or thermoregulatory responses in hot/cold environments), it is not possible to confirm the hypothesis of impaired temperature regulation. Findings from a not yet published case report from our group found altered heat and cold sensation and pain in one patient who developed neuropathy after COVID-19. Heat, in combination with the COVID-19 pandemic, has been raised as a global concern due to the interference of cooling strategies and heat risk prevention strategies, with the recommendation to stay at home [58], and might be particularly relevant for the most vulnerable populations. Among these populations, people with cognitive disorders, reduced mobility, and autonomic dysfunction might be at a higher risk of heat risk [59]. Since most of these risk factors might be present in post-COVID patients, future actions should include this category of individuals among those at risk of heat-related illnesses.

GI symptoms can be present at COVID-19 onset, alone or in combination with other symptoms. Indeed, the angiotensin-converting enzyme 2 (ACE2) receptors, where the SARS-CoV-2 spike protein binds, is highly expressed in the gut [60], and viral invasion has been suspected due to the presence of the virus in the colonic tissue and feces [61, 62]. A previous study found the prevalence of GI symptoms during the post-COVID period in nearly half of the interviewed individuals [63]. Some of these sequelae, as abnormal appetite, nausea, and diarrhea, are also part of the GI domain of the COMPASS-31 score. However, the proportion of post-COVID patients who reported at least one GI symptom was far greater in our sample (more than 3/4).

Inflammation and demyelination of the pudendal nerve are associated with the possible development of bladder incontinence and urinary symptoms, with pudendal neuropathy being a possible feature of COVID-19 as also reported in previous viral infections in animals and humans [64, 65]. In the acute phase, increased urinary frequency has been previously reported in 1/8 of male patients, and suggested to be secondary to viral cystitis due to underlying COVID-19 disease [66]. However, despite these potential mechanisms, a large cohort study found bladder dysfunction not significantly increased in the 2–5 months after COVID‐19 infection [67]. In our sample, 1/4 to 1/2 of the sample reported some sort of urinary dysfunction, including both involuntary voids and voiding difficulty. Sexual impairment, in particular erectile dysfunction, has been suggested to be possible after COVID-19 due to both endothelial and autonomic components of male erection [68, 69]. In addition, anosmia and ageusia, possible post-COVID complications, may participate in impaired sexual responses [70]. Nevertheless, it is not possible to reconduct sexual impairment only to ANS dysfunction, as psychogenic might play a fundamental role during a pandemic [71]. Our sample was characterized by similar proportions of reported sexual impairment in males and females; nevertheless, this is not surprising as sexual dysfunction in females was reported after COVID-19 disease [72].

Vision abnormalities have been reported in clinical practice by several COVID-19 and post-COVID individuals, including sore eyes and light sensitivity [73, 74]. Despite the possible influence of acute SARS-CoV-2 infection and hospitalization on ocular manifestations described in this sample, none of the participants reported eye complaints (e.g., acute conjunctivitis) during the acute phase of the disease, and an ANS involvement might be suspected. Indeed, pupil responses assessed with an automatic pupillometer were found impaired in patients recovering from COVID-19 [75]. Although this impairment might be present already during the acute phase in critically ill COVID-19 patients [76], controversial results have been found since ANS responses might be attenuated in the intensive care unit (ICU) population by ICU-related interventions, such as the administration of anesthetics, analgesics and inotropic medications that may have a prolonged effect [77]. In our sample, about one-half of the participants reported ocular impairments, and light sensitivity was the most common complaint.

This study has some limitations that need to be discussed. First, the primary ANS dysfunction evaluation was performed with a questionnaire that, despite a wide validation in different samples, suffers from possible subjective interpretation and emotional components. Indeed, the symptoms screened in the questionnaire and suggestive of ANS dysfunction are non-specific (e.g., diarrhea or urinary incontinence), and in absence of well-known condition with autonomic impairment characteristics, they might be a manifestation of disorders besides dysautonomia. Despite some studies are suggestive of an autonomic involvement after COVID-19 [9, 15], caution should be taken when considering COMPASS-31 results in this sample. The time reference for the symptoms was modified to adapt to the post-COVID period and did not affect the test–retest performance; nevertheless, some of the participants might have underrated some features that were already present before COVID-19 (e.g., GI or ocular symptoms). Second, the objective measure of OI was performed with a manual collection of heart rate and blood pressure during the active stand test, a procedure that is known to have a limited validity. Although continuous beat-to-beat monitoring should be preferred, such devices are still not common in many hospital and ambulatory services, and this data collection was performed during the first period of a dedicated ambulatory service for post-COVID manifestations. The authors are aware that objective and standard protocols for dysautonomic symptoms should be recommended; however, ANS dysfunction is often overlooked and patients are referred to dedicated centers only when persisting and severe symptoms are present. Even mild ANS impairment can be disabling and influence individuals’ quality of life.

Unfortunately, we were not able to further investigate the reported symptoms with objective techniques due to the absence of local dedicated services with the appropriate devices and protocols, and patients were referred to national centers for dysautonomic disorders. At this time, we are not able to provide findings about the diagnostic accuracy of our screening due to the absence of further follow-up and a possible selection bias (only some individuals might have had the opportunity to reach the dysautonomia center for instrumental investigations). However, although possibly overestimated, this study provides evidence of self-reported common dysautonomic manifestations in post-COVID individuals and recommends ANS screening and subsequent instrumental diagnosis as part of follow-up monitoring.

## Conclusions

This study conducted in post-COVID individuals with and without neurological manifestations revealed that most of the sample was characterized by dysautonomic symptoms, as reported with the COMPASS-31 score. Orthostatic intolerance, sudomotor, gastrointestinal, and pupillomotor abnormalities were commonly reported as complications of COVID-19. After an active stand test, about 10% of the individuals were characterized by a fall in blood pressure suggestive of orthostatic hypotension. Other dysfunctions include reduced tolerance to environmental conditions and sexual impairments. Taken together, these findings confirm the hypothesis of an ANS involvement after COVID-19, and recommend further clinical and research evaluations in post-COVID individuals with and without neurological symptoms.
